# Male partners involvement in their wives’ antenatal care and its associated factors in southern Ethiopia. A community-based cross-sectional study

**DOI:** 10.1016/j.heliyon.2024.e28276

**Published:** 2024-03-22

**Authors:** Nega Degefa, Aster Dure, Dinkalem Getahun, Zekarias Bukala, Tariku Bekelcho

**Affiliations:** a School of Nursing, College of Medicine and Health Sciences, Arba Minch University, Arba Minch, Ethiopia; b Department of Clinical Anatomy, College of Medicine and Health Sciences, Arba Minch University, Arba Minch, Ethiopia

**Keywords:** Male partner, Antenatal care, Involvement, Risk factors, Ethiopia

## Abstract

**Background:**

Involvement of male partners in antenatal care (ANC) is an effective approach to improve maternal and child health outcomes. It also enhances maternal healthcare utilization as males prevails decision-making regarding healthcare utilization in most developing countries including Ethiopia. Despite the acknowledged importance of male partners involvement, there is no research data in the study area. Therefore, the purpose of this study is to assess the status of male partners’ involvement in antenatal care and associated factors in Chencha town, which is found in southern region of Ethiopia.

**Methods:**

The study adopted a community-based cross-sectional design from April 1–30, 2022, among 560 male partners in Chencha Town. To collect data, we use a structured, pretested and interviewer-administered questionnaire. The study participants were selected using a simple random sampling method. Analysis of data was performed using the statistical package for social sciences (SPSS) version 25. Descriptive statistics including mean, frequency, and percentage were used to summarize pertinent characteristics of study participants. Both bivariable and multivariable logistic regression analyses were carried out to detect the association between the independent and outcome variables. The statistical significance was set at P < 0.05 in the final model.

**Result:**

The study found that 57% (95% CI: 53%–61%) of male partners were involved in antenatal care. Age 20 to 29 (AOR = 2.60, 95%CI:1.26, 5.37), more than secondary educational level (AOR = 2.04, 95%CI:1.08, 3.88), being government workers (AOR = 2.03, 95%CI:1.12, 3.67), exposure to information on male involvement during antenatal care (AOR = 4.37, 95%CI: 2.77, 6.91), and males’ knowledge about pregnancy danger sign (AOR = 2.55, 95%CI: 1.62, 4.02) were factors positively associated with male partner involvement in antenatal care.

**Conclusion:**

The prevalence of male partner involvement in antenatal care was relatively high, but it still needs to be improved to reach acceptable level. The involvement thrives among those aged 20–29 years, who have been exposed to information on male involvement in antenatal care, have higher education levels, government employees, and are aware of pregnancy danger signs. These factors can be used to target interventions that aim to increase male involvement in antenatal care, which helps to improve the health of both mothers and children.

## 1 Background

Reducing maternal mortality is considered a top public health priority worldwide [1]. In 2020, an estimated 287,000 women die from complications related to pregnancy and childbirth, and 95% of these deaths occur in low- and lower-middle-income countries. Sub-Saharan Africa and Southern Asia accounted for the vast majority of the deaths, with Sub-Saharan Africa alone accounting for 70% of maternal deaths [2].

In many Sub-sub-Saharan African countries, including Ethiopia, where maternal and neonatal mortality are high, women are not primary decision maker about their own health [3]. Men traditionally holds the position of family head and have notable influence over decisions regarding women's and Childrens' healthcare [4]. Involving male partner in antenatal care (ANC) delivery and child services has shown profound benefit for women and children. Studies have shown that male partner involvement in ANC increases maternal health care utilization, including institutional deliveries, postnatal check-ups, and adequate ANC [[5], [6], [7]].

Male partner involvement in antenatal care can encompass various actions, such as discussing maternal health issues, making joint decisions about pregnancy spacing, utilizing maternal health services, accompanying partners to seek antenatal care, and providing social and economic support [[8], [9], [10]].

Skilled care during pregnancy, childbirth, and the postnatal period has been shown to be effective in reducing maternal mortality in many countries [11,12].However, Ethiopia still has a high maternal mortality rate of 412 per 100,000 live births [13]. To reduce maternal mortality in Ethiopia, it is highly needed to scale up access to skilled care and to promote active male partner participation [3]. The WHO has also emphasized the promotion of active male partner participation during pregnancy, childbirth, and after birth as an important strategy [12].

Efforts to encourage male partner involvement in antenatal care have been made in both health facilities and communities [7,14]. These initiatives include couples counseling, providing educational materials to men, visiting pregnant women and their partners at home, and using mass media to reach a large audience with messages about the importance of male involvement [15,16]. While these strategies have been shown to be effective in increasing male involvement, their implementation in developing countries with high maternal and newborn mortality rates, such as Ethiopia, is still inadequate [13].

Research in Ethiopia has found that a complex interplay of factors influences male involvement in antenatal care (ANC). These factors operate across individual, interpersonal, organizational, and community levels. At the individual level, factors such as the husband's age, education, and knowledge about danger signs influence his involvement in ANC positively. Additionally, women's age at marriage were found to hinder male involvement. At the interpersonal level, differences in spousal ages impact communication and decision-making processes. Negative factors such as women's empowerment, lack of provider invitations, and unsupportive spouses impede active male involvement. Within the organizational level, the type of health facility directly impacts accessibility for male involvement. Moreover, if the spouse is a housewife, it affects the flexibility of scheduling ANC visits. At the community level, residing in rural areas introduces community-specific norms and resources that influence the extent of male involvement in ANC [[17], [18], [19], [20]].

These studies rely on women's accounts of their husbands' involvement as well as male partners' company in antenatal care (ANC) as a measure of male involvement which may not be entirely accurate thus more research is needed to better understand male partners involvement and factors affecting it.

The current study investigated male partner involvement in antenatal care using multiple dimensions of male involvement and used the male partner's own report of their involvement in antenatal care, which was more accurate. The findings from this study could be used to develop an intervention to enhance males' involvement in antenatal care, which would ultimately improve the health outcome and wellbeing of both the mother and child.

## 2 Methods and materials

### 2.1 Study setting and design

This community-based cross-sectional study was carried out from April 1–30, 2022, in Chencha town, which is in the Gamo Zone of the Southern Nation Nationalities and Peoples Region (SNNPR). The town has a total population of 111,686, of whom 51,310 are men and 60,376 women. Much of the people in Chencha are Gamo people who speak the Gamo language and are Orthodox Christians. In Chencha town, there were an estimated 60,690 people aged 5 and above who have never attended school. The town is located 37 km from Arba Minch, the seat of the Gamo Zone, and 441.4 km from Addis Ababa.

### 2.2 Study participants and data collection method

The study participants were all husbands or male partners whose wives gave birth in the last 12 months and living in Chencha town. Participants with communication difficulties and those with proven mental illnesses were not eligible to participate in the study. Data was collected using a pretested, interviewer administered, and structured questionnaire that included questions on sociodemographic characteristics, knowledge of ANC services, and knowledge on pregnancy danger signs. Trained nurses used mobile phones that ran the Android operating system and had the Open Data Kit (ODK) data collection tool pre-installed to collect the data. In cases of absence, households were visited three times to reduce non-participation.

### 2.3 Sample size determination and sampling procedure

The sample size was calculated using the single population proportion formula, which accounts for the following assumptions: 62.5% prevalence of male partners involvement from prior study [19], 5% margin of error, and 95% confidence interval. This calculation gave a sample size of 361, which was then increased by 10% to account for non-responses and multiplied by 1.5 to account for the design effect. The final sample size was 596.

**Fig. 1:**
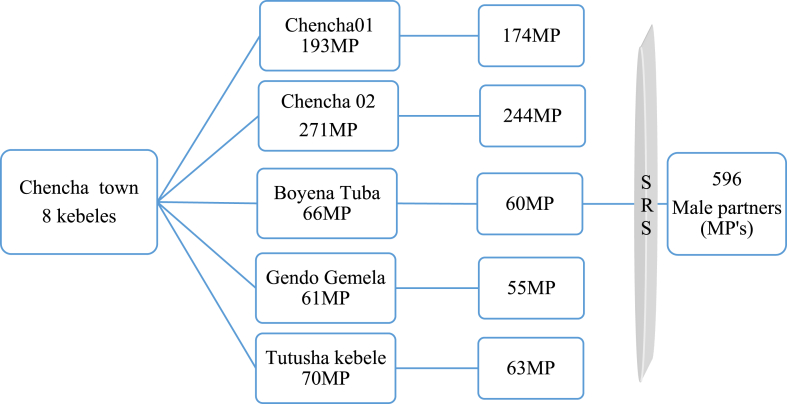
Graphic representation of sampling procedure to select male partners in Chencha town, Southern Ethiopia 2022. MP = Male Partner, SRS: Simple Random Sampling.

The first stage of the sampling process involved selecting five of the eight kebeles in Chencha town using a simple random sampling technique. In the second stage, a list of households with mothers who had given birth in the past 12 months was obtained in the selected kebeles from the health extension workers registry. This list was used to create a sampling frame, which is a list of all the households that could potentially be included in the sample. Finally, a table of random numbers was generated in SPSS using the household identification number to randomly select 596 male partners from the sampling frame proportional to the size of male partners at each kebele. The selected households were reached through their household ID and with the guidance of health extension workers (Fig. 1).

### 2.4 Study variables and measurement

**Male partner involvement:** is the main dependent variable described as the participation of husbands in the following activities; making joint plans for emergencies, delivery and postpartum care, accompanying the wife to the health facility, providing financial support, providing physical support (*like sharing household chores*), discuss maternal issues with wives, discuss the maternal issue with a healthcare provider [10,21,22]. Male partners’ responses to the activities were given a score of one [1] if performed and zero (0) if not performed. An overall scale representing the level of male partner involvement (0–6) was created for analysis. These scores were dichotomized into poor male partner involvement (0–3) and good male partner involvement [[4], [5], [6]].

**Male partners' knowledge about ANC services**: refers to the husbands' awareness of any of the formally recognized components of antenatal care. The male partners were asked to choose the components of antenatal care from the listed alternatives, and their responses were graded as to whether they had a good knowledge about components of ANC (mentioning more than half of them correctly) or a poor knowledge (mentioning fewer than half of them correctly).

**Knowledge regarding pregnancy danger signs** refer to awareness of common danger signs during pregnancy among male partners. This was determined by asking the male partners to name the signs of pregnancy they deemed to be dangerous. Therefore, answers were used to categorize male partners knowledge into either good if they properly state at least two danger signs without a prompt or poor if they are unable to do so [23].

**Exposure to messages on male partners' involvement in ANC:** describes how male partners accessed messages related to male involvement in antenatal care from different source including Television, Radio, magazines, Newspaper, and health workers. It was assessed based on husbands' reported exposure to one of these sources.

### 2.5 Data processing and analysis

The data were collected using mobile phones supporting the android operating system preinstalled with the open data kit (ODK), a mobile data collection platform. The collected data was sent to the cloud server which was aggregated and downloaded to a computer. Then, it was imported to the statistical package for social science (SPSS) version 25 for response categorization, cleaning, coding, and analysis. We performed a descriptive analysis to compile summarize pertinent data from participants, which was displayed as percentages, frequencies, means, and standard deviations in tables, graphs, texts, and charts. Binary logistic regression was carried out to determine factors associated with male partner involvement in ANC. Variables with P-values less than 0.25 were included in multivariable logistic regression analysis to adjust for possible confounding variables and identify independent predictors of male partners' involvement in the final model.

## 3 Results

A total of 560 male partners were interviewed; this led to a 94% response rate. Participants in the study ranged in age from 20 to 60 years, with a mean age of 37.10 ± 7.52. Among all participants, 281 (50.2%) practiced Protestantism, and 485 (86.6%) belonged to the Gamo ethnic group (Table 1).

**Table 1:** Sociodemographic characteristics of male partners in Chencha town, Southern Ethiopia 2022.

Variables	Category	Frequency (%)
Husbands' age (in years)	20–29 30–39 ≥40	101(18.0) 222(39.6) 237(42.3)
Religion	Orthodox Muslim Protestant	260(46.4) 19(3.40) 281(50.2)
Ethnicity	Gamo Amhara Oromo	485(86.6) 52(9.30) 23(4.10)
Education of husbands	No formal education Primary Secondary More than secondary	102(18.1) 103(18.2) 149(26.6) 206(36.8)
Number of children	1-2child 3-4 children ≥5 children	198(35.4) 209(37.3) 153(27.3)
Household's average monthly income	<4500 birr ≥4500 birr	264(47.1) 296(52.9)
Age difference with wife	≤5 years >5 years	417(74.5) 143(25.5)
Husbands' occupation	Government employed. Merchant Private worker/farmers	236(42.1) 87(15.5) 237(42.3)
Education of wife	No formal education Primary Secondary More than secondary	155(27.7) 126(22.5) 145(25.9) 134(23.9)
Occupation of wife	Housewife Government worker Private worker Merchant	289(51.6) 121(21.6) 67(12.0) 83(14.8)
Forms of marriage	Monogamous Polygamous	492(87.9) 68(12.1)

### 3.1 Prevalence of male partners' involvement in antenatal care

**Fig. 2:**
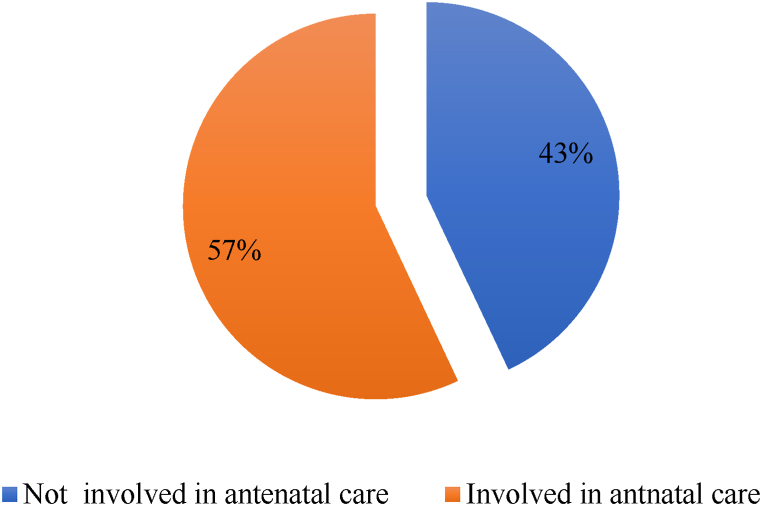
Prevalence of male partners' involvement in antenatal care in Chencha town in Southern Ethiopia, 2022.

Overall, 57% of all husbands are involved in antenatal care with a 95% CI of (53%–61%) (Fig. 2).

Most of the study participants (90.4%) reported sharing household responsibilities during pregnancy while only 35.7% of study participants accompanied their wives to ANC. During the accompanying period, most participants (80%) received health education. Most male partners (68.4%) reported they made decision emergencies with their wife's, and 57.3% stated they provided financial support during pregnancy. Only 35.0% of husbands accompany their wives to an antenatal appointment, and 39.5% discuss with a health worker about maternal issues(Table 2)

**Table 2:** Components of male partners’ involvement in antenatal care *(n = 560)* in Chencha town, Southern Ethiopia, 2022.

Variables	Response category	Frequency (%)
Male partner made a joint decision regarding emergency during pregnancy	No	177(31.6)
	Yes	383 (68.4)
Male partners provide financial support	No	239 (42.7)
	Yes	321 (57.3)
The male partner accompanies his wife to the ANC	No	360 (64.3)
	Yes	200 (35.7)
Male partners share domestic duties during pregnancy.	No	54 (9.60)
	Yes	506 (90.4)
Male partner discusses maternal issues with wives,	No	196 (35.0)
	Yes	364 (65.0)
The male partner discusses the maternal issue with the healthcare provider	No	339 (60.5)
	Yes	221 (39.5)
The male partner participated in antenatal care (ANC) health education while accompanying his wife to ANC (*n = 200*)	No Yes	40(20.0) 160(80.0)

### 3.2 Exposure to message on male partners’ involvement in ANC

Most male partners (73.2%) had access to at least one media outlet and learned about their involvement in antenatal care (ANC) from this source. Health care providers were the most common source of information about male partner involvement in ANC, reported by 76.3% of study participants, while magazines were a relatively rare source of information, reported by only 4.6% of study participants (Table 3).

**Table 3:** Distribution of access to information on male partners involvement in antenatal care among male partners in chencha town, 2022.

Variables	N (%) responses
Exposure to information on male partners involvement on ANC Yes No	410(73.2) 150(26.8)
The source of information on male partners involvement on ANC	
Television	175 (42.7%)
Radio	92(22.4)
Newspaper	45(11.0)
Magazine	19(4.6)
Health care provider	313(76.3)

### 3.3 Male partners’ knowledge of maternal antenatal care services

**Fig. 3:**
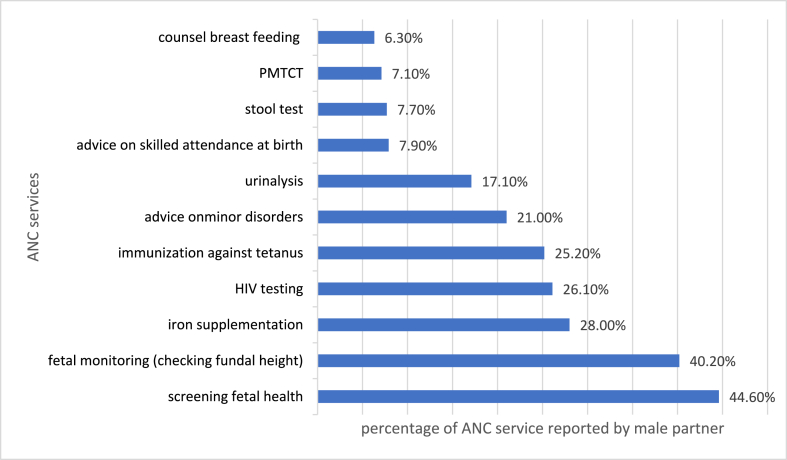
Knowledge of male partners on ANC services in Chencha town, Southern Ethiopia, 2022.

Nearly all research participants (97.5%) reported less than six antenatal care services. Fetal health screening was among the ANC service during, that male partners were most frequently mentioned (44.6%), while breastfeeding counseling (6.3%), received the least mention (Fig. 3).

### 3.4 Male partners’ knowledge of pregnancy danger sign

**Fig. 4:**
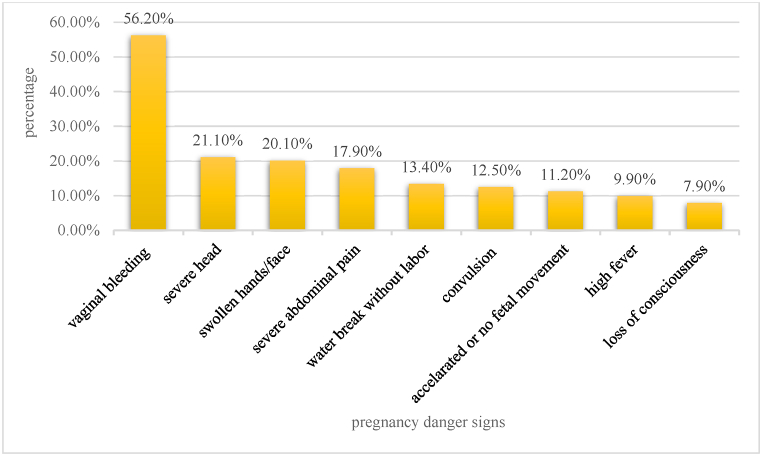
Male partner's knowledge on pregnancy danger signs in Chencha town, Southern Ethiopia, 2022.

A total of 34.8% of all participants have good knowledge or mentioned two or more pregnancy danger signs while 65.2% of all participants had poor knowledge. Study participants most frequently reported vaginal bleeding among pregnancy danger signs (56.2%), whereas loss of consciousness (7.9%), was the least frequently reported sign illustrated in (Fig. 4).

### 3.5 Factors associated with male partner involvement in antenatal care

Based on bivariable logistic regression analysis male partners' age, male partners' education, wife's education, male partners' occupation, age difference with the spouse, family's monthly income, male partners' knowledge of pregnancy danger signs, male partners' knowledge of ANC services, number of children, exposure to information on male partner involvement, and types of marriage were associated with male partners' involvement in ANC.

The multivariable logistic regression analysis includes all variables with a p-value of less than 0.25 in the bivariable analysis. Male partners' involvement in ANC was significantly associated with their age, education, occupation, exposure to information about male partner involvement, and knowledge of pregnancy danger signs (p-value 0.05).

Male partners aged 20–29 years were 2.6 times more likely than those over 40 years to participate in ANC (AOR = 2.60, 95%CI: 1.26–5.37). Husbands with at least a secondary education had two-fold increased odds of involvement in ANC compared to husbands without education (AOR = 2.04,95% CI:1.075–3.879). Compared to farmers, husbands who are government workers had twice as big odds of being involved in ANC (AOR = 2.03, 95% CI:1.12–3.67). Husbands who were informed about male involvement in ANC were 4 times more likely to participate in ANC than their counterparts (AOR = 4.37,95% CI: 2.769–6.91). Furthermore, male partners who were well-informed about pregnancy danger signs were 2.5 times more likely than their counterparts to participate in ANC (AOR = 2.55, 95% CI: 1.62–4.02) (Table 4).

**Table 4:** Bivariable and multivariable analysis of factors associated with husbands’ involvement in ANC, Chencha town, Ethiopia, 2022.

Variables	Male involvement in ANC		COR (95% CI)	AOR (95% CI)	P-value
	Yes	No			
**Husband age** 20–29 30–39 ≥40	77(76.2)	24(23.8)	3.77 (2.23, 6.37)	2.60 (1.26, 5.37)	0.010**
	133(59.9)	89(40.1)	1.76 (1.21, 2.54)	1.28 (0.79, 2.05)	0.305
	109(46.0)	128(54)	1	1	
**Male partners' Education** More than secondary Secondary education Primary education No education	149(72.3)	57(27.7)	3.73(2.27–6.15)	2.04 (1.08, 3.88)	0.029**
	82(55.0)	67(45.0)	1.75 (1.05, 2.91)	1.85 (0.97, 3.52)	0.063
	46(44.7)	57(55.3)	1.15 (0.66, 2.01)	1.38(0.71, 2.69)	0.344
	42(41.2)	60(58.8)	1	1	
**Male partners' occupation** Government employee Merchant Private/Farmer	173(73.3)	63(26.7)	3.76(2.56, 5.54)	2.03(1.12, 3.67)	0.020**
	46(52.9)	41(47.1)	1.54(0.94, 2.52)	1.23(0.69, 2.18)	0.479
	100(42.2)	137(57.8)	1	1	
**Wives' education** More than secondary Secondary education Primary education No education	97(72.4)	37(27.6)	3.63 (2.21, 5.96)	1.04(0.51, 2.09)	0.925
	89(61.4)	56(38.6)	2.20(1.39, 3.49)	1.31(0.71, 2.44)	0.392
	68(54.0)	58(46.0)	1.62(1.01, 2.61)	1.54(0.86, 2.74)	0.147
	65(41.9)	90(58.1)	1	1	
**Wives' Occupation** Merchant Government worker Private worker Housewife	48(57.8)	35(42.2)	1.42(0.87–2.32)	1.07(0.60–1.89)	0.825
	91(75.2)	30(24.8)	3.14(1.96–5.04)	1.71(0.85–3.43)	0.130
	38(56.7)	29(43.3)	1.36(0.79–2.32)	1.14(0.59–2.20)	0.701
	142(49.1)	147(50.9)	1	1	
**Age difference** ≤5 years >5 years	251(60.2)	166(38.8)	1.67(1.12, 2.44)	1.07(0.66, 1.74)	0.795
	68(47.6)	75(52.4)	1	1	
**Marriage character** Monogamous Polygamous	294(59.8)	198(40.2%)	2.55(1.51, 4.32)	1.452(0.79, 2.68)	0.234
	25(36.8)	43(63.2)	1	1	
**Children's’ number** 1–2 3-4children ≥5	137(69.2)	61(30.8)	2.73(1.76, 4.24)	1.53(0.84, 2.81)	0.166
	113(54.1)	96(45.9)	1.43(0.94, 2.18)	1.12(0.67, 1.88)	0.680
	69(45.1)	84(54.9)	1	1	
**Monthly income** ≥4500birr <4500birr	181(61.1)	115(38.9)	1.44(1.03, 2.01)	1.08(0.71, 1.66)	0.718
	138(52.3)	126(47.7)	1	1	
**Exposure to *MPI* message** Yes No	274(66.8)	136(33.2)	4.70(3.14, 7.05)	4.37(2.77, 6.91)	0.000**
	45(30.0)	105(70.0)	1	1	
**Male partners' knowledge of pregnancy danger signs** Good Poor	142(72.8)	53(27.2)	2.85(1.95, 4.15)	2.55 (1.62, 4.02)	0.000**
	177(48.5)	188(51.5)	1	1	
**Male partners' knowledge of ANC services** Good Poor	8(57.1)	6(42.9)	1.01(0.35, 2.94)	1.04(0.29, 3.61)	0.956
	311(57.0)	235(43.0)	1	1	

MPI = male partner involvement, **p < 0.05.

## 4 Discussion

This study investigated the prevalence of male partner involvement in antenatal care (ANC) and the factors associated with it. The study found that 57% of male partners were involved in ANC, which indicates that almost three out of five male partners were involved in maternal antenatal care. The study also found that male partner involvement was higher among male partners aged 20–29, those with secondary and above education, those who were government workers, those who were exposed to information about male partner involvement, and those who were aware of pregnancy danger signs.

The prevalence of male partner involvement is this study was consistent with studies conducted in India, Central Tanzania, and Ambo, Ethiopia with prevalence rates of 61% [24], 53.9% [25], and 59.9% [20] respectively. Studies carried out in Debrebirhan, Ethiopia, Myanmar, the upper east region of Ghana, and Afghanistan on the other hand, found slightly higher estimates of male partner involvement in antenatal care, at 62.2%, 64.8% [26],71.9% [27] and 69.4% [5], respectively. The mismatch could be the result of different methods used to measure the outcome variable. Unlike earlier studies which defines male accompaniment to ANC visits as male involvement, the current study measured male partners' involvement using six dimensions. The mismatch could also be driven by differences in sociocultural and some sociodemographic factors. Further large-scale studies are needed to better understand and compare these differences.

The level of male partners involvement in this study was higher than the levels observed in previous studies in Anomabo of Ghana's Central Region, Harari in Ethiopia, Bale in Ethiopia, and Indonesia. The prevalence rate in these studies were 35% [28], 19.7% [17], 41.4% [29], and 41.2% [30] respectively. The difference in male partners' involvement might be attributed to the difference in time across the studies. Another explanation for the discrepancy is that the sample size for the current study is significantly larger than that of the studies in Harar, Ghana, and Indonesia. Moreover, studies in the Harar and Bale zones were facility-based, and mothers were respondents, which may have underestimated the magnitude compared to the current study, which is a community-based and respondents are husbands.

Male partners aged 20–29 years were 2.6 times more likely to participate in antenatal care than older ones (over 40 years). Male partners who are younger are more likely to be involved in their partners' antenatal care which is consistent with previous research [19,21]. This could be explained by the fact that younger male partners have more access to and use of traditional and social media. These channels can increase their awareness of the benefits of male partners' involvement in maternal and child health, which can lead them to participate more actively. The cultural shift towards more equitable gender roles in younger generations may lead to increased male involvement in antenatal care. However, older male partners may face different barriers to participation than younger male partners. Therefore, healthcare providers and policymakers should consider tailoring antenatal care programs to engage and support older male partners. This is important to ensure that all male partners could be involved in their partner's antenatal care.

According to our finding male partners who have completed secondary education had 2 times more likely odds of better involvement in antenatal care compared to those with no education. This finding coincides with a study conducted in Nigeria [31], Nigerian Urban Region [32] and Debrebirhan Ethiopia [19].

Educational attainment among husbands plays a key role in their involvement in antenatal care. Educated partners tend to possess financial empowerment and knowledge, making them more inclined to participate in such care. In contrast, less educated male partners might encounter difficulties in discussing and involving women in maternal health decisions, possibly stemming from limited health literacy and communication skills.

This finding underscores the importance of interventions tailored to involve and empower male partners with lower educational backgrounds in antenatal care. These interventions should address possible cultural and socioeconomic obstacles to their involvement, while also fostering open communication between partners to enhance collaborative decision-making regarding maternal health matters.

This may highlight the importance of policies and programs that support education for all, particularly the disadvantaged ones in the study context.

This study found that government-employed male partners were twice more likely to be involved in antenatal care compared to private workers. This is consistent with the findings of Demise and colleagues [20]. This is because government employed male partners may have better educational status and more awareness of issues relating to maternal and child health than workers in the private sector, this is because government jobs typically require higher levels of education, and government employees may be more likely to be exposed to information about maternal and child health through their work.

Based on our findings, male partners who are knowledgeable about pregnancy danger signs were 2.6 times more likely to participate in their wife's antenatal care. The result is consistent with studies carried out in Harari public health facilities, in eastern Ethiopia as well as in Nigeria and India [17,33,34]. This would imply that being aware of danger signs encourages husbands to seek health care and participate in maternal health. This is because awareness of danger signs can lead husbands to take action to protect their partners and their babies 10.13039/100014337Furthermore, when male partners can identify danger signs, they support women to utilize health care services, particularly in emergency conditions [35].

Exposure to information related to male partner involvement in antenatal care was found to be a significant factor in this study. Thus, male partners who were exposed to information on male partners' involvement in maternal antenatal care were four times more likely to increase their involvement in antenatal care compared to male partners who were not exposed. The study, which was carried out in Central Tanzania's low-resource regions [25], supported the finding. Male partners who are exposed to information about male partners' involvement in antenatal care are more likely to have a better understanding of their role in maternal health care. This, in turn, is likely to lead to increased participation in their wives' antenatal care.

### 4.1 Strength and limitation

The inclusion of male partners or husbands as respondents was a key strength of this study, as it allowed for the collection of complete and accurate information regarding their involvement. Additionally, we used an appropriate method of analysis and an appropriate sampling approach.

This study had the following limitations: the findings in this study were subjected to a recall bias since husbands' involvement were assessed retrospectively. The participants’ response may also be affected by the presence of the data collectors. The cross-sectional nature of the study limits the researcher to make conclusions about cause and effect. The findings of the study may not be generalizable to other populations or settings. This is because the study is conducted in a small geographic area. More research is needed to develop a deeper understanding of barriers and facilitators of male partner involvement considering a qualitative approach.

## 5 Conclusion

The prevalence of male partner involvement in antenatal care was relatively high, but it still needs to be improved to reach acceptable level. The involvement thrives among those aged 20–29 years, who have been exposed to information on male involvement in antenatal care, have higher education levels, government employees, and are aware of pregnancy danger signs. These findings underscore the importance of considering a broader range of participants in efforts to further improve male partner involvement in antenatal care.

Particularly, it is imperative to extend interventions to include older husbands, individuals lacking access to information on male involvement, and those who are unaware of pregnancy danger signs. By addressing these gaps, we can further enhance male partner involvement in antenatal care, which can contribute to improved maternal health outcomes. A longitudinal study would also be useful to have a richer understanding of male partner involvement in antenatal care by tracking temporal dynamics and establishing causal relationships.

## Ethical approval and consent to participate

The study was approved by the institutional research ethical review board of the Arba Minch University College of Medicine and Health Sciences with ethical clearance number IRB/March 12, 2022. The study was implemented following the submission of an official letter from the university to the Chencha Town Health Office. Each participant confirmed their agreement to participate in the study by signing the informed consent form.

## Consent for publication

Doesn't apply.

## Availability of data and materials

The corresponding author could provide the datasets used and/or analyzed in this study upon reasonable request.

## Funding

No funding was received from any sources.

## CRediT authorship contribution statement

**Nega Degefa:** Conceptualization, Validation, Writing – original draft. **Aster Dure:** Conceptualization, Data curation, Formal analysis, Investigation. **Dinkalem Getahun:** Conceptualization, Validation, Visualization, Writing – review & editing. **Zekarias Bukala:** Visualization, Writing – review & editing. **Tariku Bekelcho:** Validation, Writing – review & editing.

## Declaration of competing interest

The authors declare that they have no known competing financial interests or personal relationships that could have appeared to influence the work reported in this paper.

## Abbreviations

ANC: antenatal care
AOR: adjusted odds ratio
CI: confidence interval
SPSS: statistical package for social science
